# A Multi-Hop Energy Neutral Clustering Algorithm for Maximizing Network Information Gathering in Energy Harvesting Wireless Sensor Networks

**DOI:** 10.3390/s16010026

**Published:** 2015-12-26

**Authors:** Liu Yang, Yinzhi Lu, Yuanchang Zhong, Xuegang Wu, Simon X. Yang

**Affiliations:** 1 College of Communication Engineering, Chongqing University, Chongqing 400044, China; yangl@cqu.edu.cn (L.Y.); xgwu@cqu.edu.cn (X.W.); 2 School of Electronic Information Engineering, Yangtze Normal University, Chongqing 408100, China; henanluyinzhi@163.com; 3 The Advanced Robotics and Intelligent Systems Laboratory, School of Engineering, University of Guelph, Guelph, ON N1G2W1, Canada; syang@uoguelph.ca

**Keywords:** wireless sensor networks, energy harvesting, energy neutral, multi-hop clustering

## Abstract

Energy resource limitation is a severe problem in traditional wireless sensor networks (WSNs) because it restricts the lifetime of network. Recently, the emergence of energy harvesting techniques has brought with them the expectation to overcome this problem. In particular, it is possible for a sensor node with energy harvesting abilities to work perpetually in an Energy Neutral state. In this paper, a Multi-hop Energy Neutral Clustering (MENC) algorithm is proposed to construct the optimal multi-hop clustering architecture in energy harvesting WSNs, with the goal of achieving perpetual network operation. All cluster heads (CHs) in the network act as routers to transmit data to base station (BS) cooperatively by a multi-hop communication method. In addition, by analyzing the energy consumption of intra- and inter-cluster data transmission, we give the energy neutrality constraints. Under these constraints, every sensor node can work in an energy neutral state, which in turn provides perpetual network operation. Furthermore, the minimum network data transmission cycle is mathematically derived using convex optimization techniques while the network information gathering is maximal. Simulation results show that our protocol can achieve perpetual network operation, so that the consistent data delivery is guaranteed. In addition, substantial improvements on the performance of network throughput are also achieved as compared to the famous traditional clustering protocol LEACH and recent energy harvesting aware clustering protocols.

## 1. Introduction

A typical wireless sensor network (WSN) [1] consists of large numbers of low-power and cheap sensor nodes with limited sensing, processing and communication abilities. When randomly deployed in sensor field, these sensor nodes can automatically self-organize into an ad hoc network [2]. Wireless sensor networks (WSNs) are widely used in many domains, such as environmental monitoring [3], target tracking [4], security surveillance [5] and disaster management [6], for the purpose of information gathering about the diverse phenomena of interest.

WSNs usually are deployed in hostile or harsh environments and work in an unattended fashion [7]. In addition, most sensor nodes in the network are driven by battery which has finite stored energy. It is inconvenient to replace or recharge the battery once its energy is exhausted. Battery life is the vital factor that restricts the lifetime of sensor nodes or network [8]. In addition, as data transmission or reception contributes the majority of energy dissipation of sensor nodes [9], an energy-efficient routing protocol is essential to conserve the energy of nodes in the network. Routing protocol has been extensively studied in the past decade and can be divided into two categories [10]: flat routing and hierarchical routing or clustering routing. All sensor nodes have the same functionalities and behave according to the same rules in flat routing [11]. The idea of clustering [12] is to group the network into several clusters, and a node in each cluster is selected to be the cluster head (CH). The responsibilities of each CH are receiving data from other normal nodes, aggregating this with its own data and forwarding the aggregated data to base station (BS). In addition, since the majority of sensor nodes only need to transmit their data to the corresponding CH for a short distance, the energy consumption of the network can be reduced effectively.

WSN technology plays a critical role for the realization of Internet of Things (IoT) [13]. In addition, Cisco has predicted that 50 billion new connections will be made in this IoT by the year of 2020 [14]. Then billions of sensor nodes will consume a significant amount of energy to execute data collecting and transmitting tasks. Once these sensors run out of energy, a lot of electronic waste will be produced which may bring a big challenge to the environment [15,16]. Because of the potential economic benefits and expected environmental sustainability, harvesting ambient energy resource from the environment to power WSNs is a promising technique [17]. Sensor nodes equipped with energy harvesting devices, such as solar panel, thermal energy harvester and microbial fuel cell, can harvest additional energy from the ambient environment [18,19]. We call this kind of sensor an *Energy Harvesting Sensor* (EH-Sensor) and refer to the WSN consists of EH-Sensors as the *Energy Harvesting Wireless Sensor Network* (EH-WSN) [20]. Such EH-WSN is very adapted to green IoT or smart city applications [21,22,23], since it can operate for extended durations with minimal installation costs.

The battery of EH-Sensors generally acts as a limited-capacity buffer of energy [24], and an energy-efficient routing protocol is also necessary to manage the energy in EH-WSNs. There are typically two types of EH-WSNs [25]. One treats harvested energy as a supplement to the battery to maximize the lifetime of WSNs. The other is using harvested energy as the only source to WSNs for perpetual network operation. In [26], a solar aware routing protocol similar to directed diffusion is proposed, which prefers to route data via solar-powered nodes. The solar energy is treated as a supplement of sensor nodes’ energy source for improving the network lifetime. In addition, the network will end inevitably at last. To achieve unlimited network lifetime when sensor nodes have the only energy source harvested from environment, the network wide energy neutral state should be guaranteed which requires each node to consume less energy than the amount of energy harvested during a certain period of time [27]. In [28], a protocol named Distributed Energy Harvesting Aware Routing (DEHAR) is proposed to find energy optimized routers in EH-WSNs. This protocol finds and maintains an energy efficient route from every source node to BS, taking into account the current energy status of the network. Sensor nodes with too little energy are excluded from these optimized routes so that they can regain the energy level through energy harvesting. Simulations show that perpetual network operation is possible for this protocol. In addition, in [18], an Energy Neutral Routing (ENR) protocol is proposed to guarantee the network wide energy neutral state in EH-WSNs. Admission controls are carried out locally at every node based on its energy budgets to admit or reject traffic routing requests, which can prevent each node from consuming more energy than harvested. In recent years, cluster-based routing for EH-WSNs has aroused wide concern. In addition, many protocols have been proposed to solve the clustering problem in single-hop EH-WSNs [20,25,29,30]. In [29], EH-Sensors are introduced to serve as the dedicated relay nodes for cluster heads (CHs), for the purposed of prolonging the network lifetime. In addition, in [20,25,30], sensor nodes have the only energy source harvested from the environment. To achieve perpetual network operation, sensor nodes’ energy harvesting ability and available energy are usually considered in these protocols when selecting CHs. To the best of our knowledge, there are fewer works about clustering in multi-hop EH-WSNs. Because of the characteristics of wireless channel, single-hop communication method may result in low energy consumption efficiency [31]. In addition, sensor nodes usually have limited communication abilities in real scenario. Thus, it is better to employ the multi-hop inter-cluster communication method so that CHs in the network can transmit data to BS cooperatively. In this paper, we concentrate on the problem of how to construct the optimal multi-hop clustering architecture in EH-WSNs, for the purpose of achieving perpetual network operation and maximizing network information gathering. Our main contributions are listed as follows:

- First, we propose a Multi-hop Energy Neutral Clustering (MENC) algorithm that ensures the network-wide energy neutral operation. MENC clusters the network with goal of providing perpetual network operation by controlling the network data transmission rate and selecting the qualified CHs based on the available energy of sensor nodes.
- Second, we construct an easy-to-deploy network architecture in which the sensor field is divided into several ring-based units, and CHs in different units compose a multi-hop routing backbone. Based on this network architecture, we analyze the energy consumption of sensor nodes for intra- and inter-cluster data transmission and then give the energy neutrality constraints. Under these constraints, every sensor node can work in an energy neutral state.
- Third, by balancing the average energy consumption of nodes in different units, we conclude a constraint formula of the number of clusters between neighbor units. Under this constraint, we can avoid packet loss problem which is caused by the limited data forwarding ability of the CHs in the unit closer to BS.
- At last, under the energy neutrality constraints, we optimize the parameters appeared in our proposed protocol through convex optimization, including the optimal number of units, minimum network data transmission cycle and number of clusters in the first unit, for the purpose of maximizing network information gathering.

The rest of this paper is organized as follows: Section 2 summarizes the existing works related to our clustering scheme. The system model and problem statement are given in Section 3. Section 4 shows our theoretical analyses about the energy neutrality constraints and the optimization of parameters. In addition, the detail of our scheme is discussed in Section 5. Section 6 is the performance evaluations of our scheme. Finally, we conclude this paper in Section 7.

## 2. Related Works

During the past few decades, clustering techniques have been extensively studied to improve the performance of WSNs. In this section, we will review the existing clustering schemes which are most related to our work. One of the most famous clustering protocols named Low Energy Adaptive Clustering Hierarchy (LEACH) is proposed in [32]. Sensor nodes in the network are grouped into several clusters, and each cluster consists of one CH and some cluster members. As the CH has responsibilities of aggregating the data from its member nodes and forwarding the aggregated data to BS, it consumes energy much faster than the member nodes. To balance the energy consumption among sensor nodes, a random rotation mechanism of CH is designed to insure that each sensor node acts as CH once during a deliberately designed time interval. However, this random CH re-selection mechanism may result in uneven distribution of CHs. Furthermore, CHs in LEACH adopt single-hop communication mode to transmit data to BS. Then the energy consumption among sensor nodes cannot be well balanced since CHs further from BS have longer data transmission distance than these closer to BS. In addition, nodes usually have limited communication abilities in real scenario. Thus, it is better to employ multi-hop communication method for inter-cluster data transmitting when designing clustering protocols for WSNs.

Hybrid Energy-Efficient Distributed clustering (HEED) is another famous clustering protocol presented in [33]. The CHs are periodically selected according to a hybrid of node’s residual energy and the intra-cluster communication cost. This CH re-selection mechanism effectively reduces the communication cost of the network and can avoid the case that more than one CH occurs in a close proximity. Furthermore, the multi-hop communication model is adopted in HEED so that CHs in the network cooperatively transmit data to BS by a multi-hop way. However, since the CHs closer to BS have heavier relay traffic and consume energy much faster than those farther from BS, the energy hole problem may occur which is not considered in HEED.

To solve the energy hole problem, unequal clustering structures have been adopted to balance the energy consumption among CHs. In [31], Chen *et al.* present an Unequal Cluster-based Routing (UCR) protocol to group the network into several clusters with unequal sizes. The CHs closer to BS have smaller cluster sizes than those farther from BS, thus they can burden less intra-cluster traffic and preserve more energy for inter-cluster data forwarding. However, the size of cluster is simply designed to decrease with the decrease of the distance between CH and BS, which needs to be analyzed and optimized. Liu *et al.* in [34] theoretically analyze the energy consumption with different cluster radius in clustering network and then give the expression of cluster radius which can maximize the network lifetime. In addition, by employing an unequal cluster radius and an alternate operation scheme between work and dormancy, a simple clustering protocol, which can mitigate the energy hole problem, is proposed. However, the network scalability is not considered in this protocol. An Energy-Balancing unequal Clustering Approach for Gradient-based routing (EBCAG) protocol is proposed in [35]. Each node maintains a gradient value which is defined as the minimum hop count to BS. In addition, based on this gradient, a ring-based network model is constructed. CHs in a ring transmit data to the heads in the neighbor ring closer to BS. By balancing the energy consumption among the CHs in different rings and minimizing the total energy consumption of the network, the size of clusters is determined in each ring. EBCAG can adapt to the expansion of the network, but how to calculate the optimal number of rings is not concerned. A protocol named Constructing Optimal Clustering Architecture (COCA) is proposed in [36]. It divides the sensor field into a number of equal-size square units. The number of clusters in each unit is obtained by balancing the total energy consumption of nodes in different units. In addition, by minimizing the total energy consumption of the network, the optimal unit size can be acquired while the optimal number of units is determined.

Though the network lifetime is greatly extended by adopting these classical schemes, the bottleneck exists as sensor nodes are energy-limited and can not be recharged in traditional WSNs. Recently, with the development of energy harvesting techniques, this problem can be effectively mitigated or eradicated. In [29], Zhang *et al.* present a clustering protocol for prolonging the lifetime of the WSNs with energy-harvesting sensors. A mixed network model is adopted in which both normal sensor nodes and the sensors with energy harvesting abilities are introduced. In addition, the energy-harvesting sensors act as dedicated relay nodes to forward data for CHs. Extensive simulations validate that the proposed protocol can efficiently achieve optimal or suboptimal solutions. An Adaptive Energy Harvesting Aware Clustering (AEHAC) protocol is proposed in [25] for EH-WSNs. Node energy state is taken into CH election mechanism. Then, a sensor node with higher energy harvesting rate or more residual energy has bigger probability to be the CH. Simulation results show that AEHAC maintains available nodes about 15% and network throughput 19% more than LEACH. In [30], a protocol named Energy Potential LEACH (EP-LEACH) is proposed to extend the traditional protocol LEACH to be suitable for EH-WSNs. An Energy Potential Function is utilized to measure sensor nodes’ energy harvesting capability. Based on this function, the probability to be CH for each node is designed. Then the nodes with extremely low energy potential will never be chosen as CHs, and nodes with energy potential above a certain threshold will be chosen as CHs with nearly equal probability. Compared with LEACH, EP-LEACH can not only extend network lifetime, but also improve the network throughput in EH-WSNs. In addition, in [20], a distributed Energy Neutral Clustering (ENC) protocol is proposed for EH-WSNs, with the goals of achieving perpetual network operation and maximizing network information gathering. A CH group (CHG) mechanism is adopted to allow that several CHs in each cluster are selected to share the heavy traffic load. This CHG mechanism can prevent the excessive energy consumption of sensors which in turn holds the network-wide energy neutral state, and then the perpetual network operation is achieved. Furthermore, an extension to ENC is proposed to maximize the amount of information gathering, and the optimal number of clusters is mathematically derived using convex optimization. Simulation results show that ENC can provide perpetual network operation and maximize network information gathering. However, the multi-hop inter-cluster communication structure is not considered in this protocol.

## 3. System Model and Problem Statement

### 3.1. Sensor and Network Model

**Figure 1:**
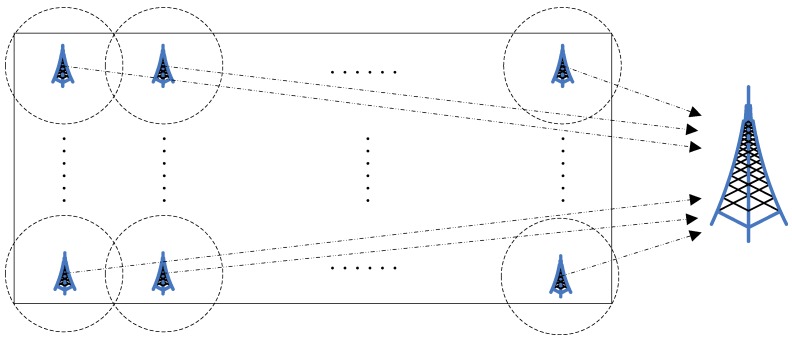
Network deployment scenario.

**Figure 2:**
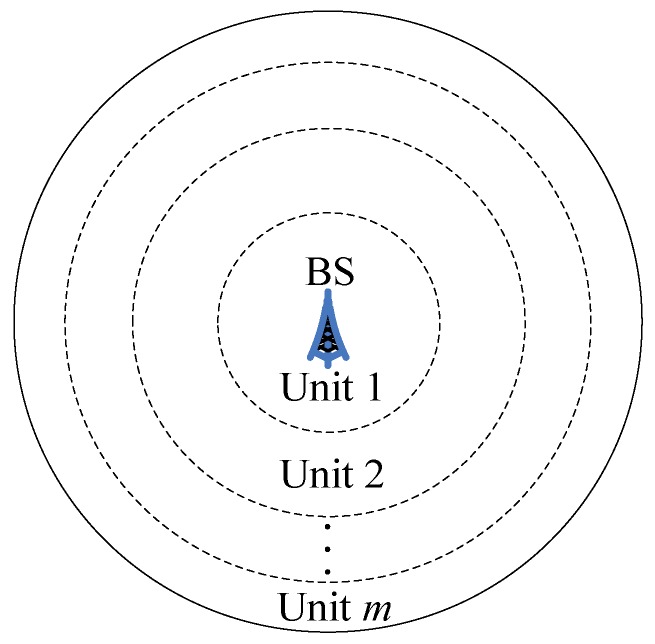
Network model.

Sensor nodes usually have limited communication ability in WSNs. When they are deployed into a large-scale sensor field, multiple BSs are necessary for the purpose of information gathering, as shown in Figure 1. In addition, for the sensor nodes distributed within a relatively smaller circular area covered by a BS, as shown in Figure 2, they can self-organize into a subnet to collect and transmit information independently. In this paper, we solve the clustering problem for such a subnet consisting of the EH-Sensors. In addition, these EH-Sensors are uniformly deployed in a circular sensor field with dense *ρ*. The BS is located at the center of this field. Let *S* denote the area of this field. We divide the circular sensor field into *m* concentric ring-based units with equal area *S*/*m*. CHs in different units cooperatively transmit data to BS by a multi-hop communication method during the data transmission process. Each CH in unit *i* (*i* > 1) selects a routing head from the CHs in unit *i*-1, and it only needs to transmit data to this routing head. Every CH in the first unit can transmit data to BS directly.

For the development of our protocol, we make several assumptions about the sensors as follows:

- All EH-Sensors are homogeneous and have the same ability to harvest energy from the ambient environment;
- Each EH-Sensor is stationary or nearly stationary after deployed in the sensor field;
- Each EH-Sensor can estimate the distance to the transmitter according to the received signal strength indicator (RSSI), if the transmitting power is known in advance;
- Each EH-Sensor can adjust its communication power level according to the distance to the expected destination;
- If an EH-Sensor serves as a CH, it compresses each member node’s data with a fixed compression ratio *a*.

### 3.2. Radio Model

Making realistic modeling of radio wave propagation between low-power sensing devices is greatly challenging. Based on the previous discussions about the radio model [36,37,38,39], two modes are adopted to analyze this work: the free space model and two-ray ground propagation model [40]. The free space model assumes that in the ideal propagation condition, there is only one clear, unobstructed line-of-sight path between the transmitter and receiver. However, two-ray ground propagation model predicts path loss when the signal received consists of the line-of-sight component and multi path component formed predominately by a single ground reflected wave. Two-ray ground propagation model is reasonably accurate for predicting the large-scale signal strength over a long transmission distance. In addition, for a shorter transmission distance, free space model gives a better prediction. Then, a distance threshold *d*_0_ is introduced to determine which model should be adopted. If the distance between the transmitter and receiver is shorter than this threshold, we adopt the free space model; otherwise, we adopt the two-ray ground propagation model.

At the transmitter, the energy spent to transmit a *k*-bit packet can be expressed as follows [38]:

(1) $$E_{Tx}(k,d)=\left\{ \begin{array}{l} kE_{elec}+k\varepsilon_{fs}d^{2}\;d<d_{0}\\ kE_{elec}+k\varepsilon_{amp}d^{4}\;d>d_{0} \end{array}\right.$$

where *d* is the distance between the transmitter and receiver; *E_elec_* is the amount of energy spent for a one-bit packet by the transmitting or receiving circuit; *ε_fs_* and *ε_amp_* are the amplifier characteristic constants corresponding to the free-space propagation model and two-ray ground reflection model, respectively; and *d*_0_ is the distance threshold which can be calculated as follows [39]:

(2) d0=εfsεamp

At the receiver, the amount of energy spent for receiving a *k*-bit packet can be calculated as follows [20]:

(3) $$E_{Rx}(k)=kE_{elec}+k\left(\frac{1}{a}-1\right)E_{DA}$$

where (1/*a*-1)*E_DA_* is the amount of energy spent to aggregate one bit of data and *a* is data compression ratio within the range of (0,1], when *a* = 1 means no compression.

As concentric ring-based network model is adopted in this paper, we assume that each CH has a short inter-cluster data transmission distance and employs free-space propagation model for its inter-cluster communication.

### 3.3. Problem Statement

Achieving perpetual network operation and maximizing network information gathering are among the central concerns when designing clustering protocols for EH-WSNs. By guaranteeing that every EH-Sensor node can work in an energy neutral state, the perpetual network operation can be achieved. This is easy to study in single-hop cluster-based routing schemes, since each CH only needs to handle the intra-cluster traffic. However, it is more complex when adopting multi-hop communication model in clustered EH-WSNs because each CH has both intra- and inter-cluster traffic. Moreover, for the purpose of maximizing network information gathering, all CHs should independently transmit data to BS at their fastest rate in single-hop clustering. However, this is not suitable for multi-hop clustering since the data from the CHs farther from BS has to be relayed by the ones closer to BS. If CHs farther from BS have faster data transmission rates than the ones closer to BS, the data from these CHs can not be immediately forwarded by the ones closer to BS that may reduce the performance of real-time systems. Next, we theoretically analyze the problem of how to achieve perpetual network operation and maximum network information gathering in a multi-hop clustered EH-WSN.

## 4. Theoretical Analyses

In this section, through theory analysis about the energy consumption of nodes for data transmitting, we first give the energy neutrality constrains. Under these constraints, each node can work in an energy neutral state. In addition, then, we conclude a constraint formula of the number of clusters between neighbor units by balancing the average energy consumption of nodes in different units. At last, under the energy neutrality constrains, we optimize the parameters in our protocol for the purpose of maximizing network information gathering.

### 4.1. Energy Neutrality Constrains

We assume that each sensor node transmits its sensing data to the corresponding CH or BS periodically, and sensor nodes in different units may have different data transmission cycles. Let *T_i_* (1 ≤ *i* ≤ *m*) be the data transmission cycle of nodes in unit *i*, and *q* be the length of data packet transmitted to CH or BS by a node during each cycle. As aforementioned, to achieve perpetual network operation, each sensor node should work in an energy neutral state. That is, during each cycle, a sensor node should consume less energy than the total amount of energy which contains the accumulated residual energy before this cycle and the additional energy resource harvested from the ambient environment in the current cycle. Since CHs consume energy much faster than normal nodes, all sensor nodes within a cluster should take turns serving as the CH to distribute the energy consumption. Let *n_i_* (1 ≤ *i* ≤ *m*) be the average number of sensor nodes per cluster in unit *i*, and then each node in this unit can serve as the CH once every *n_i_* cycles. To maintain every sensor node in unit *i* in an energy neutral state, the energy consumption of nodes should satisfy the following constraints:

(4) $$Ecm_{i}\leq T_{i}P_{e}\left(i=1,2,\cdots ,m\right)$$

(5) $$Ech_{i}\leq n_{i}T_{i}P_{e}-\left(n_{i}-1\right)Ecm_{i}\left(i=1,2,\cdots ,m\right)$$

where *Ecm_i_* is the average amount of energy consumed by a non-cluster head node in unit *i* to transmit data to its corresponding CH; *Ech_i_* is the average amount of energy consumed by a CH in unit *i* to receive data from its member nodes, aggregate this with its own data and transmit the aggregated data to its routing head or BS; *P_e_* is the energy harvesting rate; *T_i_* is the data transmission cycle of the nodes in unit *i*; and *n_i_* is the average number of sensor nodes per cluster in unit *i*.

Equation (4) guarantees that a normal node consumes less energy than the amount of energy it can harvest during each cycle. Equation (5) guarantees that a CH consumes less energy than the total amount of energy including the accumulated residual energy before the current cycle and the energy harvested during this cycle.

*Ecm_i_* and *Ech_i_* can be computed by the following equations respectively:

(6) $$Ecm_{i}=q\left(E_{elec}+\varepsilon_{fs}r_{i}^{2}\right)$$

(7) $$Ech_{i}=\frac{\rho \left(m-i\right)\frac{S}{m}aq}{c_{i}}\left(2E_{elec}+\varepsilon_{fs}d_{i}^{2}\right)+\left(n_{i}-1\right)qE_{elec}+n_{i}q\left(\frac{1}{a}-1\right)E_{DA}+n_{i}aq\left(E_{elec}+\varepsilon_{fs}d_{i}^{2}\right)$$

where *q* is data packet length of each node; *r_i_* is the average distance between a non-cluster head node and the CH within a cluster in unit *i*; *ρ* is node density; *m* is the number of units; *S* is the sensor field size; *a* is data aggregation ratio; *c_i_* is the number of clusters in unit *i*; *n_i_* is average number of sensor nodes per cluster in unit *i*; and *d_i_* is the average distance between a CH in unit *i* and its routing head or BS.

On the right-hand side of Equation (7), the first term is the average energy consumed by a CH in unit *i* to receive and forward the inter-cluster data traffic. In addition, the rest three terms are the average energy consumed by a CH in unit *i* to receive, aggregate and forward the intra-cluster data traffic, respectively.

As the average number of sensor nodes per cluster in unit *i* (1 ≤ *i* ≤ *m*) is *n_i_*, and *ρ* is node density, then the average cluster radius of a CH in unit *i* can be calculated by

(8) $$Rc_{i}=\sqrt{\frac{n_{i}}{\rho \pi }}$$

and by adopting the same method in [41], we can calculate *r_i_* as follows:

(9) $$r_{i}=\frac{\rho }{n_{i}}\int_{\theta =0}^{2\pi }\int_{r=0}^{Rc_{i}}r^{2}drd\theta =\frac{2}{3}\sqrt{\frac{n_{i}}{\rho \pi }}$$

where *ρ* is node density; *n_i_* is average number of sensor nodes per cluster in unit *i*; and *n_i_*/*ρ* represents the average area covered by a cluster in unit *i*.

The number *c_i_* of clusters in unit *i* can be expressed by the average number *n_i_* of sensor nodes per cluster in this unit, which is shown as follows:

(10) $$c_{i}=\rho \frac{S}{mn_{i}}$$

The average distance *d_i_* between a CH in unit *i* and its routing head or BS can be calculated by

(11) $$d_{i}=d_{i\to BS}-d_{i-1\to BS}$$

where $d_{i\to BS}$ is the average distance between nodes in unit *i* and BS, which can be calculated as follows:

(12) $$d_{i\to BS}=\frac{m}{S}\int_{\theta =0}^{2\pi }\int_{r=R_{i-1}}^{R_{i}}r^{2}drd\theta =\frac{2\pi m}{3S}\left(R_{i}^{3}-R_{i-1}^{3}\right)$$

where *m* is the number of units; *S* is sensor field size; and *R_i_* is the outer radius of unit *i* which is computed as follows:

(13) $$R_{i}=\sqrt{i\frac{S}{m\pi }}$$

And then we can recalculate $d_{i\to BS}$ as follows:

(14) $$d_{i\to BS}=\frac{2}{3}\left(i\sqrt{\frac{iS}{m\pi }}-\left(i-1\right)\sqrt{\frac{\left(i-1\right)S}{m\pi }}\right)$$

For a CH in unit *i*, it consumes much more energy than a normal node in the same unit. That is, *Ecm_i_* < *Ech_i_*. Thus, Equation (4) is redundant and Equation (5) can effectively restrict each node to work in an energy neutral state. In addition, based on Equation (5), the energy neutrality constraint for the sensor nodes in unit *i* can be re-expressed as follows:

(15) $$\frac{Ech_{i}\text{+}\left(n_{i}-1\right)Ecm_{i}}{n_{i}P_{e}}\leq T_{i}\left(i=1,2,\cdots ,m\right)$$

Let *Eav_i_* represent the average energy consumption of nodes per cycle in unit *i*, and it can be expressed as follows:

(16) $$Eav_{i}\text{=}\frac{\left(n_{i}-1\right)Ecm_{i}+Ech_{i}}{n_{i}}$$

Then Equation (15) can be simplified as follows:

(17) $$\frac{Eav_{i}}{P_{e}}\leq T_{i}\left(i=1,2,\cdots ,m\right)$$

To maximize the network information gathering, nodes in each unit should transmit data to BS at the fastest rate. That is, the data transmission cycle should be minimized. As a result, the energy neutrality constrain for the nodes in unit *i* can be updated as follows:

(18) $$\frac{Eav_{i}}{P_{e}}=T_{i}\left(i=1,2,\cdots ,m\right)$$

### 4.2. Balancing the Average Energy Consumption of Nodes in Different Units

For any sensor node in unit *i* (1 ≤ *i* ≤ m), if Equation (18) is satisfied, this node can work in an energy neutral state. Then we explore how to maximize network information gathering under the energy neutrality constraint. Assuming that the information can be continuously collected by each sensor node in the network, then the following question is to determine the information transmission cycle of a sensor node based on its available energy.

For a CH in the unit closer to BS, once received data from the CH farther from BS, it should immediately forward this data if it has enough energy, otherwise, it discards this data. This is reasonable in terms of the following two aspects: First, real-time transmission of data is more meaningful than non-real-time transmission; Second, as sensor nodes in the unit closer to BS have heavier relay traffic and consume energy much faster than the ones in the unit farther from BS, then if a CH has not enough energy to forward the received data, it still has not enough energy to forward this data over time because it should give priority to forward the newly received data after it harvested some energy. In addition, then, if *T_j_*< *T_i_* (*j* >* i*), packet loss problem will occur since CHs in the unit closer to BS have lower data transmission rate and can not timely forward data from the CHs in the unit farther from BS. In addition, if *T_j_*> *T_i_* (*j* >* i*), the network information gathering can not be maximized as the nodes in the unit farther from BS have the potential to enhance their data transmission rate. Thus, to solve these problems, the constraint *T_i_*= *T_j_*(*i* ≠ *j*) should be satisfied. In addition, according to Equation (18), the average energy consumption of nodes in different units should be balanced to guarantee this constraint. That is

(19) $$Eav_{i\text{+}1}\text{=}Eav_{i}\left(i=1,2,\cdots ,m-1\right)$$

where *Eav_i_* and *Eav_i_*_+1_ are the average energy consumption of nodes per cycle in unit *i* and *i* + 1, respectively.

Combining Equations (6), (7), (9), (10), (16) and (19), we have

(20) $$\left(\psi +\phi \frac{1}{c_{i+1}c_{i}}\right)\left(c_{i}-c_{i+1}\right)=\omega$$

where *c_i_* and *c_i_*_+1_ are the number of clusters in unit *i* and *i* + 1; and parameters *φ*, *ψ* and *ω* are shown respectively as follows:

(21) $$\psi =\frac{2mqE_{elec}}{\rho S}$$

(22) $$\phi =q\varepsilon_{fs}\frac{4S}{9\pi m}$$

(23) $$\omega =\left(m-i+1\right)aq\varepsilon_{fs}d_{i}^{2}-\left(m-i\right)aq\varepsilon_{fs}d_{i+1}^{2}+2aqE_{elec}$$

where *m* is the number of units in the network; *q* is data packet length of each node; *ρ* is node density; *S* is sensor field size; *a* is data aggregation ratio and *d_i_* is the average distance between a CH in unit *i* and its routing head or BS.

According to Equations (11) and (14), we can have

(24) $$d_{i+1}<d_{i}\left(i=1,2,\cdots ,m-1\right)$$

which implies that the CHs in the unit closer to BS have longer inter-cluster communication distance. Thus, we have *ω*> 0 according to Equation (23). In addition, based on Equations (20−22), we have

(25) $$c_{i+1}<c_{i}\left(i=1,2,\cdots ,m-1\right)$$

which shows that for the purpose of balancing the average energy consumption of nodes in different units, clusters in the unit closer to BS should strictly more than that in the unit farther from BS.

As a result, if Equation (20) is met, the average energy consumption of nodes in different units can be balanced. In addition, then the constraint *T_i_*= *T_j_*(*i* ≠ *j*) can be satisfied. Let *T* denote the network data transmission cycle, then we have

(26) $$T_{i}=T_{j}=T\left(i,j=1,2,\cdots ,m\right)$$

According to Equations (11), (14) and (21)–(23), to calculate *φ*, *ψ* and *ω* in Equation (20), the number of units *m* should be determined. In addition, we will find the optimal value of *m* in the following subsection.

### 4.3. Maximizing Network Information Gathering

When designing clustering protocols for EH-WSNs, another main objective is to maximize network information gathering, which needs to minimize the network data transmission cycle *T*. According to Equations (18) and (26), we can realize this objective by minimizing the average energy consumption *Eav*_1_ of nodes in the first unit. Combining Equations (6), (7), (9)–(11), (14) and (16), *Eav*_1_ is calculated as follows:

(27) $$Eav_{1}=\frac{-2qE_{elec}m}{\rho S}c_{1}+\frac{4Sq\varepsilon_{fs}}{9\pi m}\frac{1}{c_{1}}+2aqE_{elec}m+\Delta$$

where *q* is data packet length of each node; *m* is the number of units; *ρ* is node density; *S* is sensor field size; *c*_1_ is the number of clusters in the first unit; *a* is data aggregation ratio; and Δ is a constant to *m* and *c*_1, _and it is expressed as follows:

(28) $$\Delta =aq\varepsilon_{fs}\frac{4S}{9\pi }+q\left(\frac{1}{a}-1\right)E_{DA}-q\varepsilon_{fs}\frac{4}{9\rho \pi }+\left(2-a\right)qE_{elec}$$

According to Equation (27), the derivative of *Eav*_1_ with respect to *c*_1_ can be calculated by the following equation:

(29) $$\frac{d}{dc_{1}}\left(Eav_{1}\right)=\frac{-2qE_{elec}m}{\rho S}-\frac{4Sq\varepsilon_{fs}}{9\pi m}\frac{1}{c_{1}^{2}}$$

From Equation (29), we find *d*(*Eav*_1_)/*dc*_1_ < 0, which means *c*_1_ should be maximized to minimize *Eav*_1_. In addition, when all sensor nodes in the first unit are selected as CHs, *c*_1_ reaches the maximum value which can be expressed as follows:

(30) $$c_{1}=\rho \frac{S}{m}$$

where *S* is sensor field size; *ρ* is node density and *m* is the number of units.

According to Equations (27) and (30), we can find *d*(*Eav*_1_)/*dm* > 0, which means the number of units *m* should be minimized to minimize *Eav*_1_.

As aforementioned, we adopt the free-space propagation model for the inter-cluster communication. Then the maximum inter-cluster data transmission distance *d_max_* should meet the following constraint:

(31) $$d_{\max}\leq d_{0}$$

where *d*_0_ is the distance threshold that calculated by Equation (2).

We define unit size as the difference between the outer radius and inner radius of this unit. In addition, for any unit *i* (1 ≤ *i* ≤ *m*), its inner radius is equal to the outer radius of unit *i* - 1, then its size *Ru_i_* can be expressed as follows:

(32) $$Ru_{i}=R_{i}-R_{i-1}$$

where *R_i_* is the outer radius of unit *i* (*R*_0_ = 0).

Combining Equations (13) and (32), the size *Ru*_i_ of unit *i* (1 ≤ *i* ≤ *m*) can be recalculated as follows:

(33) $$Ru_{i}=\left(\sqrt{i}-\sqrt{i-1}\right)\sqrt{\frac{S}{m\pi }}$$

where *S* is sensor field size and *m* is the number of units.

From Equation (33), we can find that the unit closer to BS has larger size than the one father from BS. For a CH in unit *i* (1 < *i* ≤ *m*), we assume it has the tendency to select its routing head from the CHs in unit *i*-1 toward the direction of BS. Then for the CHs in unit *i* (1 ≤ *i* ≤ *m*), the one nearest the outer edge may have the longest inter-cluster data transmission distance $d_{\max}^{\left(i\right)}$, which is estimated as follows:

(34) $$d_{\max}^{\left(i\right)}=Ru_{i}+Ru_{i-1}$$

where *Ru_i_*_-1_ and *Ru_i_* are the size of unit *i* - 1 and *i* (*Ru*_0_ = 0).

The maximum inter-cluster data transmission distance *d_max_* can be expressed as follows:

(35) $$d_{\max}=\max\left\{d_{\max}^{\left(i\right)}|i=1,2,\cdots ,m\right\}=d_{\max}^{\left(2\right)}$$

Combing Equations (33)–(35), we can recalculate *d_max_* as follows:

(36) $$d_{\max}=\sqrt{\frac{2S}{m\pi }}$$

where *S* is sensor field size and *m* is the number of units.

And based on Equations (2), (31) and (36), we have

(37) $$m\geq \frac{2S\varepsilon_{amp}}{\pi \varepsilon_{fs}}$$

As aforementioned, for the purpose of minimizing *Eav*_1_, the optimal value of *m* should be equal to its minimum value, which is expressed as follows:

(38) $$m=\left\lceil \frac{2S\varepsilon_{amp}}{\pi \varepsilon_{fs}}\right\rceil$$

As a result, the number of clusters in each unit can be determined based on Equations (11), (14), (20)–(23), (30) and (38). In addition, if Equations (18), (26−28), (30) and (38) are satisfied simultaneously, the network information gathering is maximized since the network data transmission cycle *T* reaches the minimum value which is expressed as follows:

(39) $$T\text{=}\frac{\left(2\left\lceil \frac{2S\varepsilon_{amp}}{\pi \varepsilon_{fs}}\right\rceil -1\right)aqE_{elec}+aq\varepsilon_{fs}\frac{4S}{9\pi }+q\left(\frac{1}{a}-1\right)E_{DA}}{P_{e}}$$

where *m* is the optimal number of units; *a* is data aggregation ratio; *q* is data packet length of each node; *S* is sensor field size and *P_e_* is energy harvesting rate.

Note that, only the number *c*_1_ of clusters in the first unit is the optimal value since it is obtained by minimizing the average energy consumption *Eav*_1_ of nodes in this unit. In addition, the numbers of clusters in other units are acquired by balancing the average energy consumption of nodes in different units. Thus, if nodes in each unit transmit data based on the minimum network data transmission cycle *T* calculated by Equation (39), only the data transmission rate of nodes in the first unit is maximized.

## 5. The Detail of Our Protocol

**Figure 3:**
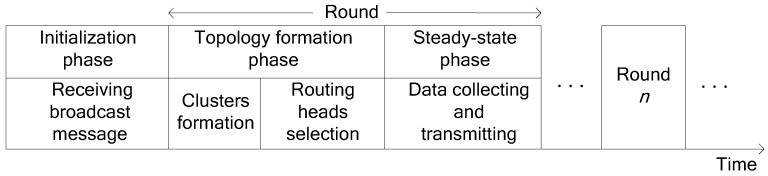
Time line denoting the procedure of MENC.

In this section, we will describe the detail of our protocol. We call it Multi-hop Energy Neutral Clustering (MENC). As shown in Figure 3, the procedure of MENC consists of a one-time initialization phase and many repeated rounds that can be further divided into topology formation phase and steady-state phase. The duration of each round is just the minimum network data transmission cycle *T*. During each round, sensor nodes in every unit will be re-grouped into several clusters. In addition, a cluster consists of one CH and some cluster member nodes. Since a cluster member node consumes less energy than it can harvest during each round, its accumulated residual energy will reach a certain threshold after several rounds (energy accumulation cycle), and then it has the eligibility to be selected as a CH. Since the units closer to BS have more clusters, the nodes in these units have shorter energy accumulation cycle and smaller energy threshold. For any node in unit *i* (1 ≤ *i* ≤ *m*), its energy accumulation cycle is *n_i__·_T*, since it serves as the CH once every *n_i_* rounds. In addition, based on Equations (16), (18) and (26), its energy threshold *ETh_i_* can be calculated as follows:

(40) $$ETh_{i}=Ech_{i}=n_{i}TP_{e}-\left(n_{i}-1\right)Ecm_{i}$$

where *Ech_i_* is the average energy consumed by a node in unit *i* when serving as a CH; *n_i_* is the average number of sensor nodes per cluster in unit *i*; *T* is the minimum network data transmission cycle; *P_e_* is energy harvesting rate; and *Ecm_i_* is the average energy consumed by a non-cluster head node in unit *i* to transmit data to its corresponding CH.

In the initialization phase, BS broadcasts a message with different power level *Lev_i_* (1 ≤ *i* ≤ *m*) corresponding to the outer radius *R_i_* (1 ≤ *i* ≤ *m*) of each unit. In addition, this message includes the minimum network data transmission cycle *T*, optimal number *m* of units, cluster numbers *c_i_* (1 ≤ *i* ≤ *m*), average numbers *n_i_* (1 ≤ *i* ≤ *m*) of sensor nodes per cluster and energy thresholds *ETh_i_* (1 ≤ *i* ≤ *m*). A sensor node only receives the message broadcasted by BS with the lowest power level and determines which unit it belongs to based on this message. In addition, this node also estimates its distance to BS according to the received signal strength. CHs in each unit will be selected in the topology formation phase, and each one in unit *i* (1 < *i* ≤ *m*) will select a routing head from the CHs in unit *i* - 1. All CHs act as a routing backbone and cooperatively transmit data to BS. Based on this backbone, each sensor node will begin to transmit its sensing data to BS during the steady-state phase. To avoid the conflict of signals on the wireless channel, each CH creates an individual time division multiple access (TDMA) schedule for its cluster members. Every cluster member node transmits its sensing data to the CH in its own time slot and goes dormant during other time slots. Once a CH has received the data from its member nodes, it aggregates this with its own data by adopting data compression techniques and then transmits the data to its routing head or BS using a randomly selected code division multiple access (CDMA) code.

**Figure 4:**
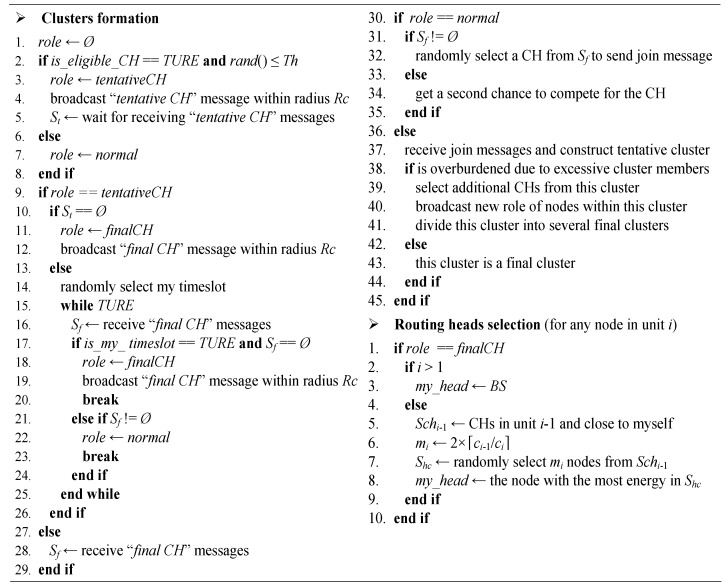
Topology formation phase pseudo code in Multi-hop Energy Neutral Clustering (MENC).

In the rest of this section, we will give the detail of topology formation phase which includes clusters formation and routing heads selection. In addition, the pseudo code of the topology formation phase is shown in Figure 4.

### 5.1. Clusters Formation

To construct the cluster topology, four steps are performed sequentially. In addition, they are tentative CHs selection, final CHs contention, tentative clusters formation and final clusters formation.

Our CH selection mechanism is similar to that adopted in LEACH. At the beginning of each round, a sensor node which has the eligibility to be a CH randomly chooses a number within range [0,1]. In addition, if this number is less than a threshold, this node will serve as a CH in the current round. The threshold *Th_i,j_* for any sensor node *j* in unit *i* can be expressed as follows:

(41) $$Th_{i,j}=\{\begin{array}{cc} \frac{p_{i}}{1-p_{i}\left(r\mathrm{mod}\frac{1}{p_{i}}\right)} & \text{if }j\in G\\ 0 & \text{otherwise} \end{array}$$

where *p_i_* is the desired percentage of CHs in unit *i*; *r* is the current round and *G* is the set that consists of the sensor nodes which have eligibility to be CHs.

If node *j* meets the following two conditions, it can be classified into set *G*: First, it has not been a CH during the last 1/*p_i_* rounds; Second, its accumulated energy is more than the energy threshold *ETh_i_*. Since a sensor node in unit *i* serves as a CH once every *n_i_* rounds, then *p_i_* can be calculated by

(42) $$p_{i}=\frac{1}{n_{i}}$$

For a CH in unit *i*, its cluster radius is *Rc_i_* which is calculated by Equation (8). To avoid the case that two CHs in the same unit may be within each other’s cluster radius, these nodes successfully selected as CHs keep the CH state tentatively (tentative CH) and will further compete for the final CHs. Each tentative CH first broadcasts “*tentative CH*” message (including node ID, unit number and CH status) within the corresponding cluster radius. In addition, then, by receiving this kind of messages broadcasted by other CHs in the same unit and storing these messages into set *S_t_*, each tentative CH knows its neighbor CHs within its cluster radius. If a tentative CH has no neighbor CHs, it will immediately announce itself to be a final CH by broadcasting the “*final CH*” message (including node ID, unit number and CH status) within its cluster radius. In addition, then, its neighbor sensor nodes know its successful election by receiving this message and storing it into set *S_f_*. If a tentative CH has several neighbor CHs, it will announce itself to be a final CH in a randomly selected time slot. In addition, once hearing the announcement, its neighbor CHs will abandon the declaration to be a final CH and return to the normal state.

After all final CHs are determined, each normal sensor node will choose a CH in the same unit to join cluster. If a normal node has received several “*final CH*” messages, it randomly selects one CH to join tentative cluster; Otherwise, this normal node (isolate node) will be given another chance to compete for the CH. If the residual energy of an isolate node is more than the corresponding energy threshold, this node will broadcast a “*final CH*” message in a randomly selected time slot. In addition, an isolate node which has received one or more “*final CH*” messages before it broadcasts this kind of message, it will give up broadcasting the “*final CH*” message and randomly select one message broadcaster to join tentative cluster; otherwise, it will be a final CH.

For a tentative cluster in unit *i*, it becomes the final cluster automatically if there are no more than *n_i_* sensor nodes in it. However, if this cluster contains more than *n_i_* sensor nodes, the corresponding CH may have no enough energy to burden the intra-cluster data traffic. To avoid this problem, such a tentative cluster should be divided into several final clusters with no more than *n_i_* sensor nodes in each of them. For the CH in this tentative cluster, firstly, it selects enough additional CHs (we refer to them as the newly selected CHs) with more residual energy from the normal nodes within the same cluster; Secondly, it chooses the nearest *n_i_* - 1 normal nodes as its final member nodes; Thirdly, it randomly selects less than *n_i_* normal nodes as cluster member nodes for each newly selected CH; At last, it broadcasts a message including the role (normal node or newly selected CH) of each node and the corresponding CH of each normal node within the cluster.

Then after all final clusters are formed, the CHs in these clusters will select their own routing heads using the scheme described below.

### 5.2. Routing Heads Selection

Since CHs in the first unit are very close to BS, they transmit data to BS directly. As shown in Equation (25), the units closer to BS have more CHs than the ones farther from BS. Let *m_i_*= 2×⌈*c_i_*_-1_/*c_i_*⌉ (1 < *i* ≤ *m*). For any CH *j* in unit *i* (expressed as *ch_i,j_*), it randomly selects *m_i_* CHs from set *Sch_i_*_-1_ as its routing head candidates and stores them into set *S_hc_*. In addition, the candidate with the most residual energy will be selected as its final routing head. This random candidate selection mechanism can mitigate the unbalance of inter-cluster traffic. The set *Sch_i_*_-1_ (1 < *i* ≤ *m*) is expressed as follows:

(43) $$Sch_{i-1}=\{ch_{i\text{-}1,k}|dis\left(ch_{i,j},ch_{i\text{-}1,k}\right)\leq dis\left(ch_{i,j},BS\right)-d_{i-2\to BS},ch_{i\text{-}1,k}\text{ is any CH }k\text{ in unit }i-1\}$$

where *dis*(*ch_i,j_*, *ch_i_*_-1*,k*_) is the distance between CH *ch_i,j_* and *ch_i_*_-1*,k*_; *dis*(*ch_i,j_*, BS) is the distance from CH *ch_i,j_* to BS and $d_{i-2\to BS}$ is the average distance from the nodes in unit *i*-2 to BS ($d_{0\to BS}$= 0).

## 6. Performance Evaluations

In this section, we will evaluate the performance of our Multi-hop Energy Neutral Clustering (MENC) algorithm via simulations performed on MATLAB platform. We first give the performance comparison among energy harvesting aware routing protocols mentioned above, as shown in Table 1. In addition, then we evaluate the performance of our protocol by comparing it with the most related ones. 

Since we are devoted to constructing the optimal multi-hop clustering architecture for EH-WSNs in this paper, we will evaluate the performance of our protocol by comparing it with all the energy harvesting aware clustering protocols mentioned in Table 1. In addition, the CH selection mechanism in our protocol is related to that in LEACH, we will evaluate the performance of MENC by comparing it with LEACH as well. As the protocol proposed in [29] is not named, we call it P-29 for simplicity.

**Table 1:** Performance comparison among energy harvesting aware routing protocols.

Routing Protocol	Routing Category	Harvested Energy Attribute	Network Attribute	Is Perpetual Network Operation Achieved
[26]	Flat	Supplement	Single-hop	Not achieved
DEHAR	Flat	Only source	Multi-hop	Achieved
ENR	Flat	Only source	Single-hop	Achieved
[29]	Clustering	Supplement	Single-hop	Not achieved
AEHAC	Clustering	Only source	Single-hop	Not achieved
EP-LEACH	Clustering	Only source	Single-hop	Not achieved
ENC	Clustering	Only source	Single-hop	Achieved
MENC	Clustering	Only source	Multi-hop	Achieved

### 6.1. Parameters Setup

The parameters used throughout the simulations are listed in Table 2.

**Table 2:** Simulation parameters.

Parameters	Values
Packet size *q* (bits)	1000
*E_elec_* (nJ/bit)	50
*E_Tx_* (nJ/bit)	50
*E_Rx_* (nJ/bit)	50
*ε_fs_* (pJ/bit/m^2^)	10
*E_DA_* (nJ/bit/message)	5
Node density *ρ* (node/100m^2^)	1
Compression ratio *a*	0.2
Energy harvesting rate *P_e_* (J/h)	0.5

Since all sensor nodes in the first unit act as CHs and transmit data to BS directly in MENC, let sensor nodes in the first unit in LEACH, AEHAC, EP-LEACH, ENC and P-29 adopt the same method. Besides, the probability *P_C_* of a node (not in the first unit) to be selected as a CH in LEACH is computed by

(44) $$P_{C}\text{=}\frac{1}{n-S/m}\sum_{i=2}^{m}c_{i}$$

where *m* is the optimal number of units in MENC; *S* is the area of sensor field; *c_i_* is the number of clusters in unit *i* and *n* is the total number of sensor nodes in the network.

Equation (44) indicates that the probability of a node to be selected as a CH in LEACH is equal to the average probability of a node to be CH in MENC.

The probability of a node to be a Center Node (CN) in ENC is also computed by Equation (44), to keep consistency of the number of clusters between MENC and ENC. In addition, the initial probability of a node to be the CH in AEHAC is calculated by Equation (44) in the following simulations. Moreover, we set the number of clusters *N_C_* predefined in P-29 to be the total number of clusters in MENC.

### 6.2. Simulation Results

In this section, we simulate a total of 500 rounds of data transmission. In addition, we suppose that before transmitting data to the destination in each round, every CH checks whether it has enough energy according to the distance to the destination. If a CH has no enough energy for data transmission, a cluster failure will occur and this CH will discard the data packet in the current round. In addition, as the CH in each cluster has a dedicated relay node (RN) in protocol P-29, cluster failure means a RN has no enough energy to relay data. We adopt four metrics to evaluate the performance of MENC, including Average Cluster Failure Times per Round (ACFTR), Average Network Throughput per Round (ANTR), Average Network Throughout per Second (ANTS) and Average Energy Consumption per Round (AECR). Here we define ANTR as the average number of data packets successfully collected by BS per round, and ANTS is the average number of data packets successfully collected by BS per second.

We first evaluate the performance of MENC under the case that sensor nodes are uniformly deployed in a circular sensor field with area 4×10^4^ m^2^, and the data transmission cycle ranges from 0.4·*T* to 2·*T*. Here *T* is the minimum network data transmission cycle calculated by Equation (39). As node density *ρ* is 1 node/100 m^2^, the total number of sensor nodes is 400. In addition, according to Equation (38), the number of units is 4.

**Figure 5:**
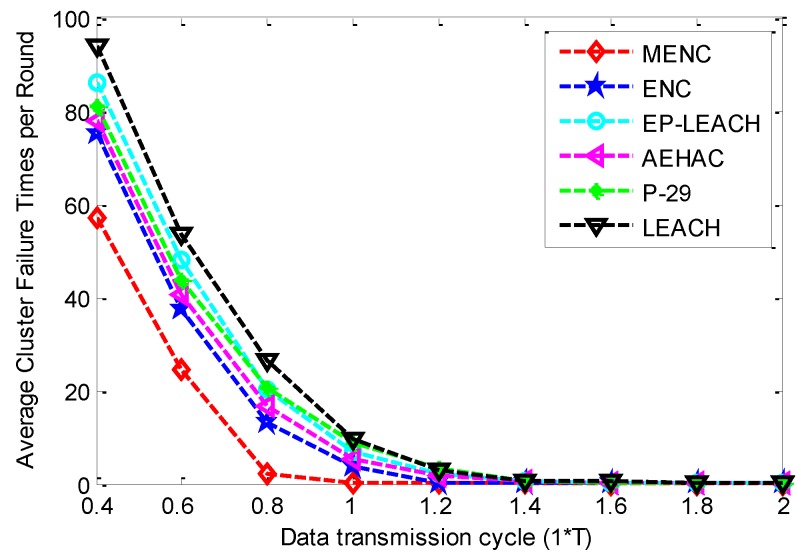
The comparison of ACFTR among MENC, ENC, EP-LEACH, AEHAC, P-29 and LEACH for different data transmission cycles.

Figure 5 gives the comparison of Average Cluster Failure Times per Round (ACFTR) among the six clustering protocols for different data transmission cycles. This figure shows that with the increase of data transmission cycle, the ACFTR of all the six protocols decrease until to the minimum value 0. This is because that each CH or RN has more time to harvest energy from the ambient environment with the increase of data transmission cycle. In addition, this figure also shows that MENC outperforms other five protocols in terms of ACFTR. This owes to the energy neutrality constraint considered in MENC. Under this constraint, a sensor node is not eligible to be CH until it harvests enough energy from the environment, and the number of sensor nodes within a cluster is not beyond the expectation of the CH. The protocol ENC adopts a Cluster Head Group (CHG) mechanism to allow that several sensor nodes serve as the CH in turn within a cluster to share the traffic load. It has smaller ACFTR than EP-LEACH, AEHAC, P-29 and LEACH. However, a sensor node with less energy may be selected as a member of CHG, which increases the probability of cluster failure. Then ENC has poorer performance than MENC in terms of ACFTR. With the increase of data transmission cycle, EP-LEACH firstly has bigger ACFTR than P-29, and then it has smaller ACFTR than P-29. This is because that the protocol P-29 selects a dedicated RN for the CH within each cluster so that the traffic load within a cluster is shared by RN and CH. Then P-29 has smaller probability of cluster failure than EP-LEACH when sensor nodes have not enough time to harvest energy from the environment. In addition, when sensor nodes have more time to harvest energy, EP-LEACH has less ACFTR than P-29 since sensor nodes with more available energy have bigger probability to be the CH in EP-LEACH. In AEHAC, an energy threshold is introduced to ensure that sensor nodes with too little energy switch to sleep mode for saving energy. Moreover, the sensor nodes with more available energy have bigger probability to be the CH. Thus, AEHAC has smaller ACFTR than EP-LEACH and P-29.

**Figure 6:**
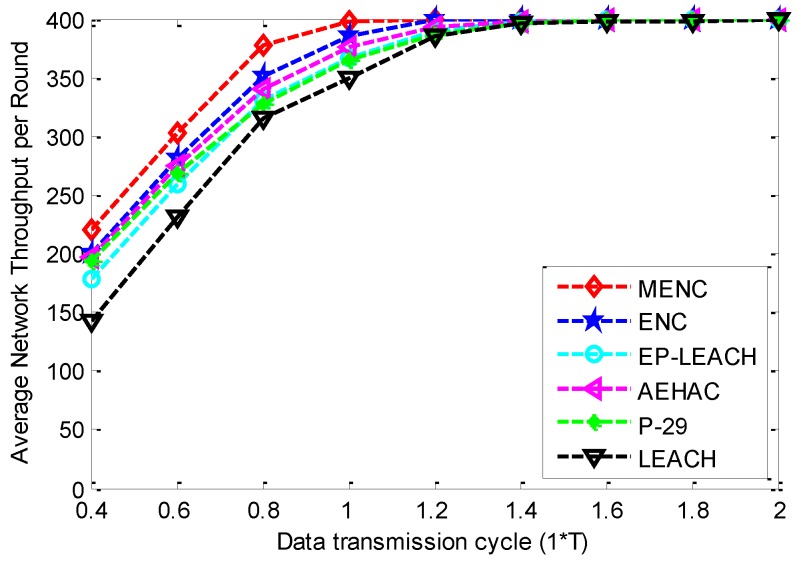
The comparison of ANTR among MENC, ENC, EP-LEACH, AEHAC, P-29 and LEACH for different data transmission cycles.

The comparison of Average Network Throughput per Round (ANTR) among the six clustering protocols for different data transmission cycles is given in Figure 6. From this figure, we can see that with the increase of data transmission cycle, the ANTR of all the six protocols increase until to the maximum value. This is because the ACFTR decreases with the increase of data transmission cycle for the six protocols. In addition, we can also find that before the ANTR of the six protocols reach the maximum value, MENC has bigger ANTR than other five protocols. This is because that MENC has the smallest ACFTR compared with ENC, EP-LEACH, AEHAC, P-29 and LEACH. In addition, MENC has higher energy efficiency than these protocols since it adopts multi-hop communication method for inter-cluster data transmission.

**Figure 7:**
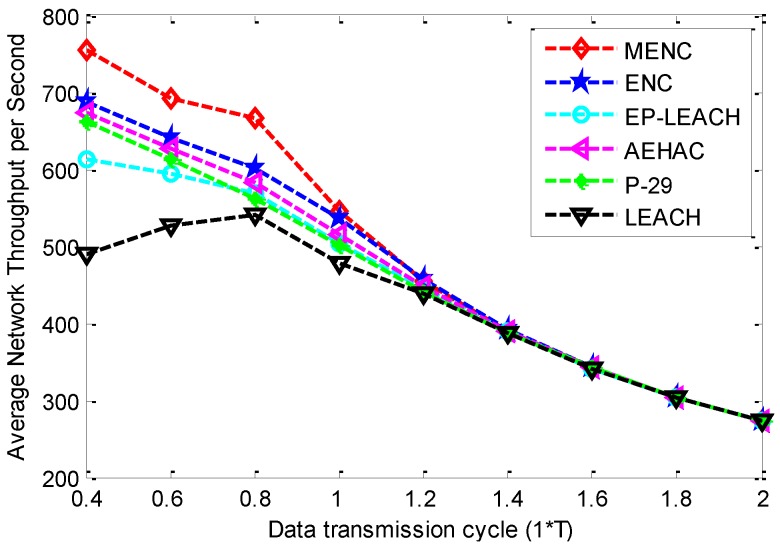
The comparison of ANTS among MENC, ENC, EP-LEACH, AEHAC, P-29 and LEACH for different data transmission cycles.

The comparison of Average Network Throughput per Second (ANTS) among the six clustering protocols for different data transmission cycles is shown in Figure 7. From this figure, we can see that with the increase of data transmission cycle, the ANTS of MENC, ENC EP-LEACH, AEHAC and P-29 decrease, and that of LEACH increases firstly and then decreases. This phenomenon can be explained as follows: with the increase of data transmission cycle, the ANTR increases for all the six protocols. In addition, LEACH has faster increase rate of ANTR than that of data transmission cycle when data transmission cycle is smaller than 0.8 *T*. This figure also shows that MENC has bigger ANTS than other protocols, and the difference of ANTS among the six protocols tends to be zero when data transmission cycle increases. This is because MENC has bigger ANTR than other protocols, and the ANTR tends to be the maximum value for the six protocols with the increase of data transmission cycle.

Based on the above results, we can conclude that *T* is the optimal data transmission cycle in MENC. This is because that on the one hand, the network-wide energy neutral state can be well guaranteed when data transmission cycle is equal to or bigger than *T*, since the ACFTR is nearly zero and the ANTR is nearly maximal according to Figure 5 and Figure 6; On the other hand, as the ANTS decreases rapidly with the increase of data transmission cycle, the ANTS can be maximized on the premise of guaranteeing perpetual network operation when data transmission cycle is equal to *T*.

**Figure 8:**
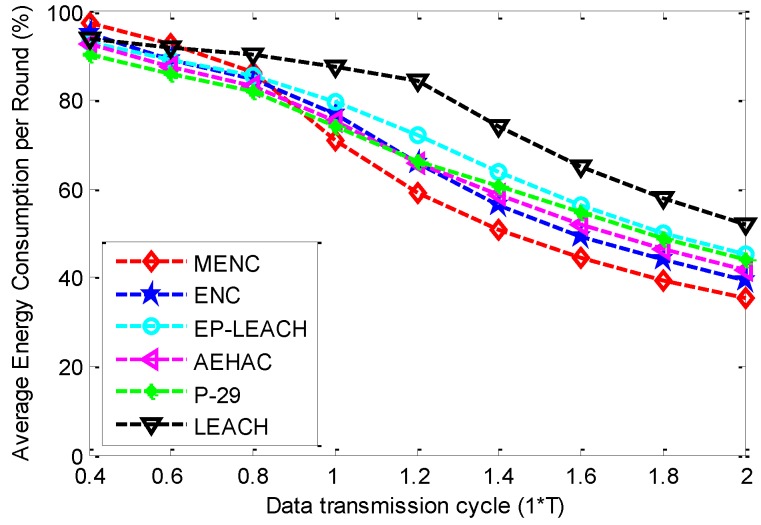
The comparison of AECR among MENC, ENC, EP-LEACH, AEHAC, P-29 and LEACH for different data transmission cycles.

In order to observe the energy consumption of nodes in the network, we give the Average Energy Consumption per Round (AECR) for different data transmission cycles, as shown in Figure 8. From this figure, we can find that the AECR of the six protocols have a downward trend with the increase of data transmission cycle. This is because more energy can be harvested by each node with the increase of data transmission cycle. When data transmission is smaller than 0.8 *T*, MENC has bigger AECR than other five protocols since it has fewer cluster failure times. In addition, according to Figure 5, Figure 6 and Figure 8, we can conclude that MENC has the highest energy efficiency among the six protocols. This can be explained as follows: when data transmission cycle is bigger than 1.2 *T*, MENC has smaller AECR than other five protocols and nearly the same ACFTR and ANTR with other protocols; In addition, a node has higher energy efficiency if it consumes less energy for transmitting the same amount of data. Since multi-hop communication method is adopted in MENC and other five protocols adopt the single-hop one, this conclusion is also in accordance with the case that multi-hop communication has higher energy efficiency than single-hop one.

In the following simulations, we evaluate the performance of MENC under the case that the sensor field size *S* ranges from 2 × 10^4^ m^2^ to 6 × 10^4^ m^2^, and the data transmission cycle is equal to *T* which is calculated by Equation (39).

**Figure 9:**
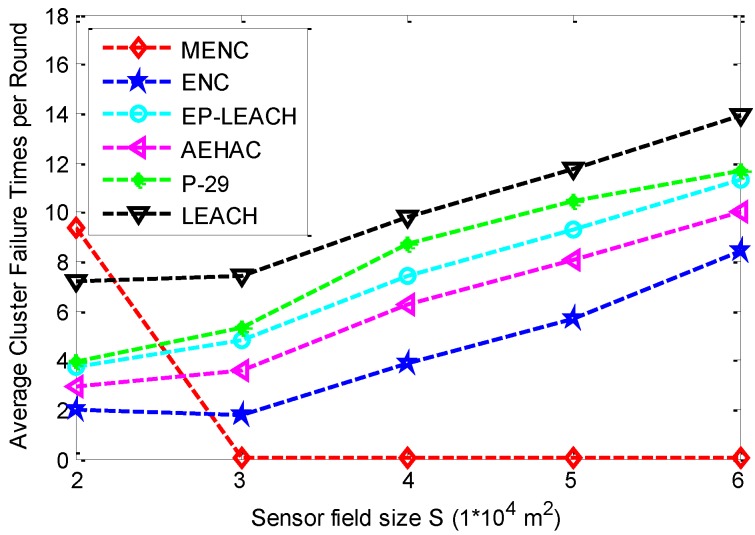
The comparison of ACFTR among MENC, ENC, EP-LEACH, AEHAC, P-29 and LEACH for different sensor field sizes.

The comparison of ACFTR among the six clustering protocols for different sensor field sizes is given in Figure 9. This figure shows that the ACFTR of MENC is bigger than other five protocols when *S* is equal to 2 × 10^4^ m^2^, and it tends to be zero when *S* increases. This is because multi-hop communication method is adopted in MENC. In addition, MENC can work well in large-scale networks as sensor nodes need not to transmit data to BS directly for a long distance. However, when the network size is small, data from the nodes farther from BS still has to be relayed by the ones closer to BS that may result in performance degradation of the protocol. From this figure, we can also find that the ACFTR of ENC, EP-LEACH, AEHAC, P-29 and LEACH increase with the increase of sensor field size *S*. This is because single-hop communication model is adopted by these five protocols. In addition, with the increase of *S*, CHs or RNs have longer data transmission distance to BS which increases the probability of cluster failure.

**Figure 10:**
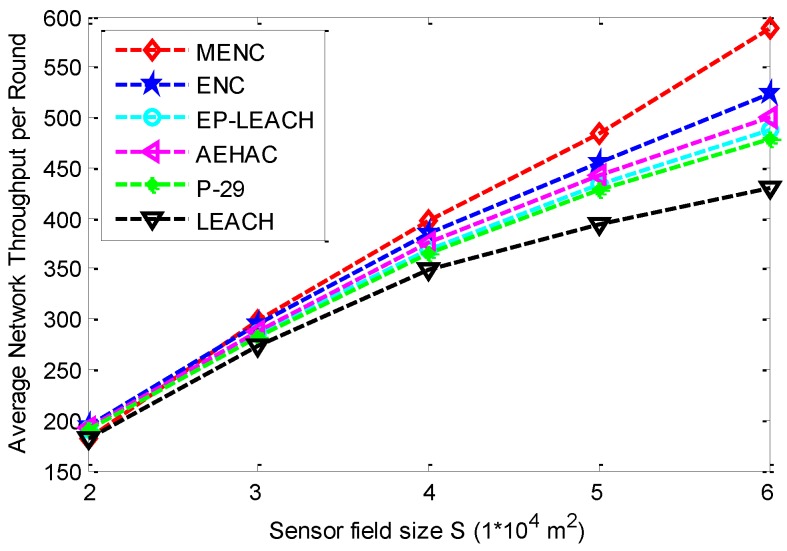
The comparison of ANTR among MENC, ENC, EP-LEACH, AEHAC, P-29 and LEACH for different sensor field sizes.

Figure 10 gives the comparison of ANTR among the six clustering protocols for different sensor field sizes. The ANTR of all the six protocols increase with the increase of sensor field size *S*. This is because the total number of sensor nodes is proportional to *S*. More data can be collected and transmitted to BS with the increase of *S*. In addition, when *S* is equal to 2 × 10^4^ m^2^, MENC has smaller ANTR than other five protocols since its ACFTR is the smallest among these protocols.

## 7. Conclusions

In this paper, we present a Multi-hop Energy Neutral Clustering (MENC) algorithm to construct the optimal multi-hop clustering architecture in EH-WSNs, for the purpose of achieving perpetual network operation and maximizing network information gathering. In MENC, the sensor field is divided into several units with equal size, and sensor nodes in each unit are grouped into some clusters. By analyzing the energy consumption of intra- and inter-cluster data transmission, we give the energy neutrality constraints which guarantee that each sensor node can work perpetually with consistent data delivery. Furthermore, to maximize network information gathering, we optimize the parameters appeared in our proposed protocol using convex optimization techniques, and these parameters include the optimal number of units, number of clusters in each unit and minimum network data transmission cycle. Extensive simulation results verify that MENC can avoid the case of cluster failures and guarantee the perpetual operation of the network. In addition, compared to LEACH and recent energy harvesting aware clustering protocols, MENC is more energy-efficient since it can provide larger network throughput.
